# Supplementary material for: MAURIVAX: A Vaccination Campaign Project in a Hospital Environment for Patients Affected by Autoimmune Diseases and Adult Primary Immunodeficiencies
Source: Vaccines (Basel). 2023 Oct 11;11(10):1579. doi: 10.3390/vaccines11101579 (PMC10610841; doi:10.3390/vaccines11101579)
Supplement: Supplementary file 1 [file vaccines-11-01579-s001.zip › vaccines-2624398-supplementary.pdf]

Supplementary Material

Table S1. Doses of vaccines administered, divided for each type of vaccine and stratified by degree of immunosuppression and disease.

**Autoimmune disease-vaccinated patients**	**Type of immunosuppressive therapy**	**N° (%) patients**	**N°of administered doses**					
			**Shingrix**	**Prevenar 13**	**Pneumovax**	**Bexsero**	**Menveo**	**Hiberix**
**Not taking immunosuppressive therapy**		9 (25%)	18	2	1	2	1	0
**Taking Immunosuppressive therapy**	**Combined high grade**	16 (44,4%)	32	2	0	2	1	0
	**Single high grade**	8 (22,2%)	16	0	0	2	1	0
	**Single low/moderate grade**	3 (8,3%)	6	1	0	2	1	1
**PID (confirmed or highly suggestive)**		**N° (%) patients**	**Shingrix**	**Prevnar-13**	**Pneumovax**	**Baxero**	**Menveo**	**Hiberix**
Highly suspected common variable immunodeficiency being diagnosed		**2** (28,5%)	4	0	0	0	0	0
Confirmed Common Variable Immunodeficiency (CVID)		**2** (28,5%)	4	1	1	0	0	0
Confirmed IgG deficiency		**2** (28,5%)	4	0	0	2	0	0
Confirmed X-linked agammaglobulinemia		**1** (14,2%)	2	0	0	0	0	0
Organ transplant (liver)		**1**	2	1	0	0	0	0
Severe asthma		**1**	2	0	0	0	0	
**Total**		**45**	90	7	2	10	4	1
